# Isoflurane impairs oogenesis through germ cell apoptosis in *C. elegans*

**DOI:** 10.1038/s41598-021-93737-0

**Published:** 2021-07-14

**Authors:** Tao Zhang, Cheng Ni, Cheng Li, Pan Lu, Dan Chen, Yuanlin Dong, Johnathan R. Whetstine, Yiying Zhang, Zhongcong Xie

**Affiliations:** 1 Department of Anesthesiology, Tenth People’s Hospital of Tongji University, Shanghai, 200072 China; 2 Department of Anesthesiology, National Cancer Center/National Clinical Research Center for Cancer/Cancer Hospital, Chinese Academy of Medical Sciences and Peking Union Medical College, Beijing, 100021 China; 3 Department of Anesthesiology and Perioperative Medicine, Translational Research Institute of Brain and Brain-Like Intelligence, Shanghai Fourth People’s Hospital, School of Medicine, Tongji University, Shanghai, 200434 China; 4 Geriatric Anesthesia Research Unit, Department of Anesthesia, Critical Care and Pain Medicine, Massachusetts General Hospital and Harvard Medical School, 149 13th Street, Room 4310, Charlestown, MA 02129-2060 USA; 5 Department of Anesthesiology, Xi’an Jiaotong University Second Affiliated Hospital, Xi’an, 710004 China; 6 Cancer Epigenetics Institute, Fox Chase Cancer Center, Philadelphia, PA 19111 USA; 7 Cancer Signaling and Epigenetics Program, Institute for Cancer Research, Fox Chase Cancer Center, Philadelphia, PA 19111 USA

**Keywords:** Apoptosis, Toxicology, Oogenesis

## Abstract

Anesthetic isoflurane has been reported to induce toxicity. However, the effects of isoflurane on fecundity remain largely unknown. We established a system in *C. elegans* to investigate the effects of isoflurane on oogenesis. Synchronized L4 stage *C. elegans* were treated with 7% isoflurane for 4 h. Dead cells, ROS, embryos, and unfertilized eggs laid by hermaphrodites were measured by fluorescence imaging and counting. The *C. elegans* with losses of *ced-3, cep-1, abl-1*, male *C. elegans,* and oxidative stress inhibitor N-acetyl-cysteine were used in the interaction studies. We found that isoflurane decreased the numbers of embryos and unfertilized eggs and increased the levels of dead cells and ROS in *C. elegans*. The isoflurane-induced impairment of oogenesis was associated with *abl-1*, *ced-3,* but not *cep-1*. N-acetyl-cysteine attenuated the isoflurane-induced impairment of oogenesis in *C. elegans*. Mating with male *C. elegans* did not attenuate the isoflurane-induced changes in oogenesis. These findings suggest that isoflurane may impair oogenesis through *abl-1-* and *ced-3-*associated, but not *cep-1*-associated, germ cell apoptosis and oxidative stress, pending further investigation. These studies will promote more research to determine the potential effects of anesthesia on fecundity.

## Introduction

Studies in rodents and cultured cells have suggested that anesthetic could induce neurotoxicity and cytotoxicity^1^. However, whether anesthetic can also cause impairment in fecundity in *C. elegans* remains largely to be determined. Previous studies show an association between general anesthesia and lower pregnancy success rates from in vitro fertilization^2,3^, while others demonstrate the opposite^4–6^. The effect of anesthetics on embryos and unfertilized eggs, however, remains mostly unknown. Therefore, we investigated the impact of anesthetic isoflurane on fecundity in *C. elegans* by looking at oogenesis through assessing the numbers of embryos and unfertilized eggs in *C. elegans* following isoflurane treatment.

Short exposures to clinical concentrations of nitrous oxide, isoflurane, and halothane do not affect early embryonic growth in mice^7^. However, administration of isoflurane at concentrations similar to those used during human oocyte recovery for in vitro fertilization inhibits mouse embryo development^8^. Moreover, isoflurane can induce mitochondrial dysfunction, apoptosis, and oxidative stress^9–11^, impairing oogenesis and reproduction^12^.

Germ cell apoptosis is an integral part of oogenesis in animals and humans^13^. In *C. elegans*, physiological germ cell apoptosis occurs in normal oogenesis, reducing the number of oocytes^14^. Specifically, germ cells, which differentiate into sperm and oocytes, can undergo apoptotic cell death, maintaining germ cell homeostasis. Physiological germ cell apoptosis serves as a “nurse cell” model to eliminate half of all oogenesis germ cells. Both DNA damage-associated^12^ and environmental stress-associated germ cell apoptosis^15^ occur in *C. elegans* and are needed for oocyte maturation^14^. Thus, we set out to assess whether isoflurane could impair oogenesis and understand the underlying mechanisms in *C. elegans*.

A core pathway including the conserved proteins EGL-1/BH3, CED-9/BCL2, CED-4/APAF1, and CED-3/caspase regulates most cell death in *C. elegans*^16^. The most downstream core component of the apoptotic cell death pathway is CED-3^17^. Ras/mitogen-activated protein kinase (MAPK) signaling is a crucial regulator of physiological germ cell apoptosis^14^. DNA damage-associated germ cell apoptosis associated with ionizing irradiation is uniquely affected by CEP-1 (*C. elegans* P53-like)^18,19^. Previously, we showed that isoflurane could induce DNA damage through oxidative stress and the *p53* pathway in neuronal cells^10^. Finally, ABL-1 is essential for stress-induced germ cell apoptosis^15^.

Therefore, we used *C. elegans* with loss of *ced-3*, *cep-1,* and *abl-1* to assess the role of germline apoptosis in the effects of anesthesia on oogenesis*.* Specifically, we investigated whether DNA damage-associated germ cell apoptosis (regulated by *cep-1*) or stress-induced germ cell apoptosis (regulated by *abl-1*) contribute to the isoflurane-induced oogenesis impairment. In *C. elegans*, oxidative stress can also cause germ cell apoptosis through an *abl-1*-associated pathway^15^. We, thus, hypothesized that isoflurane impairs oogenesis by inducing oxidative stress and germ cell apoptosis in *C. elegans*.

## Results

### Isoflurane impairs oogenesis in *C. elegans*

**Figure 1:**
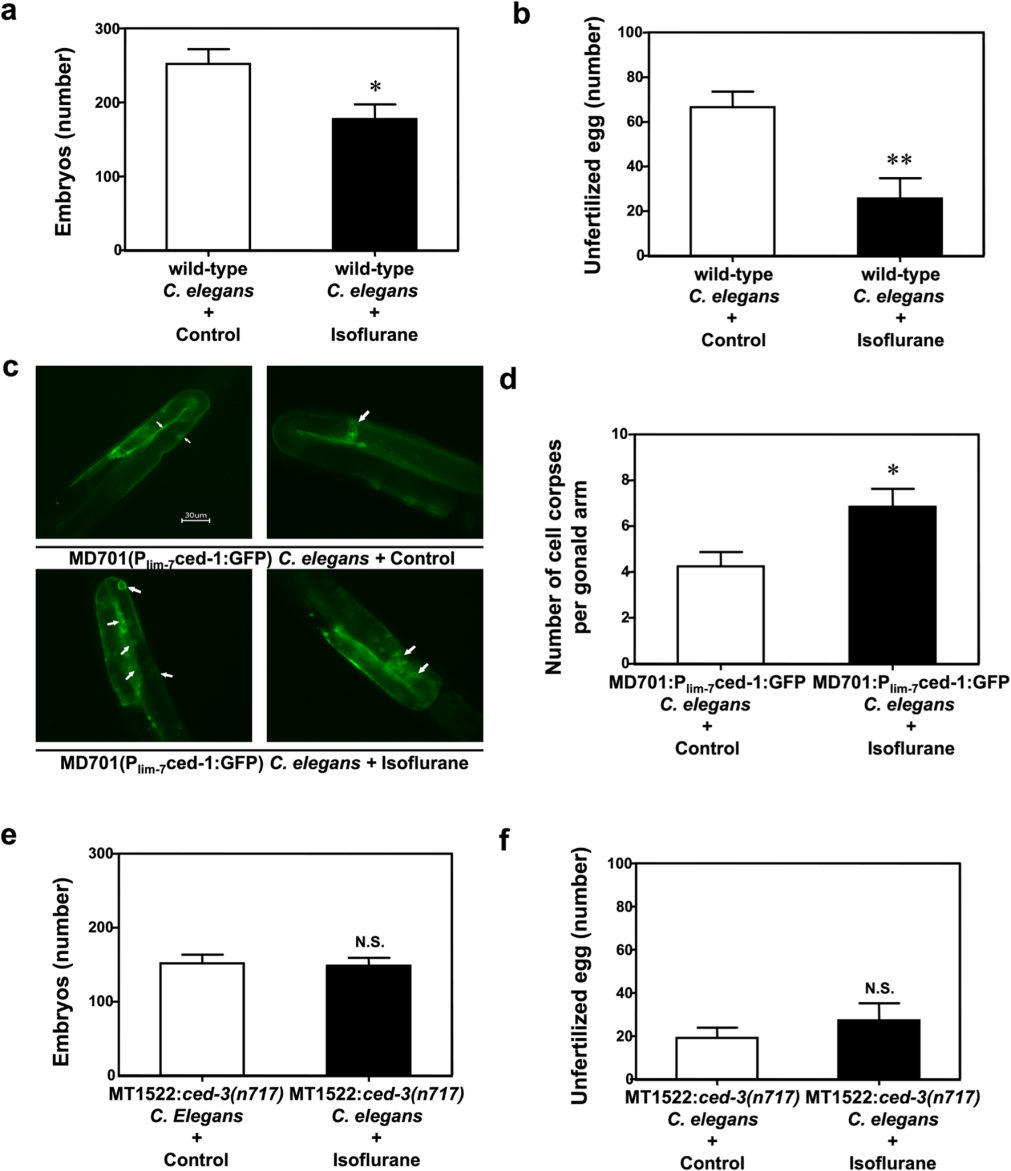
Isoflurane impaired oogenesis in *C. elegans*. (**a**) Treatment with 7% isoflurane for 4 h decreased the number of embryos produced by N2 hermaphrodites. (**b**) Treatment with 7% isoflurane for 4 h decreased the number of unfertilized eggs produced by N2 hermaphrodites. (**c**) Fluorescence microscopy of representative dead cells (at day one with 400 × magnification using z-stacking) produced by adult day-1 MD701 hermaphrodites. Arrows indicate dead cells with the green halo. Scale: 30 µm. (**d**) Quantification of dead cells produced by adult MD701 hermaphrodites at day one. Isoflurane increased the number of dead cells. (**e**) Treatment with 7% isoflurane for 4 h did not decrease the number of embryos produced by *ced-3* knockout hermaphrodites. (**f**) Treatment with 7% isoflurane for 4 h did not decrease the number of unfertilized eggs produced by *ced-3* knockout hermaphrodites. All data are presented as mean ± S.D. N = 10 hermaphrodites in each group. The Student's t-test was used to analyze the data presented in (**a**,**b**,**d**–**f**); the P values refer to the difference between the control condition and isoflurane treatment. * *P* < 0.05, ** *P* < 0.01. N2: wild-type *C. elegans*. MD701: *C. elegans* strain *bcls39 V*. MT1522: *C. elegans* strain *ced-3(n717)*.

N2 hermaphrodites were treated with 7% isoflurane (the EC50 for immobility in *C. elegans*) for 4 h. *C. elegans* hermaphrodites only produce ~ 300 progeny because of their limited number of sperms. After laying embryos, hermaphrodites produced unfertilized eggs for another 1–2 days. Thus, both embryos and unfertilized eggs were counted to evaluate the effects of isoflurane on oogenesis. Isoflurane treatment significantly reduced the number of embryos (177 ± 63 *versus* 252 ± 62, *P* = 0.031, Student’s t-test) and unfertilized eggs (26 ± 29 *versus* 67 ± 22, *P* = 0.005, Student’s t-test) in N2 hermaphrodites (Fig. 1a,b).

MD701 *C. elegans* were used for a germline apoptosis assay. Adult day 1 MD701 hermaphrodite was exposed to 7% isoflurane for 4 h, and dead cells were counted in each gonad arm under a fluorescence microscope. The number of deal cells significantly increased following the isoflurane treatment compared to the control condition (6.9 ± 3.5 *versus* 4.2 ± 2.8, *P* = 0.020, Student’s t-test) (Fig. 1c,d). We used MT1522 mutants, which have the knockout of *ced-3*, to repeat the fertilization assay. MT1522 mutants showed no significant difference in the number of embryos (Fig. 1e) and unfertilized eggs (Fig. 1f) between the isoflurane treatment and the control condition. We did not observe isoflurane-induced changes in uterus, uterine muscles, vulva, vulva muscles, and local neuropils (data not shown).

### Isoflurane impairs oogenesis via a *cep-1*-independent mechanism

**Figure 2:**
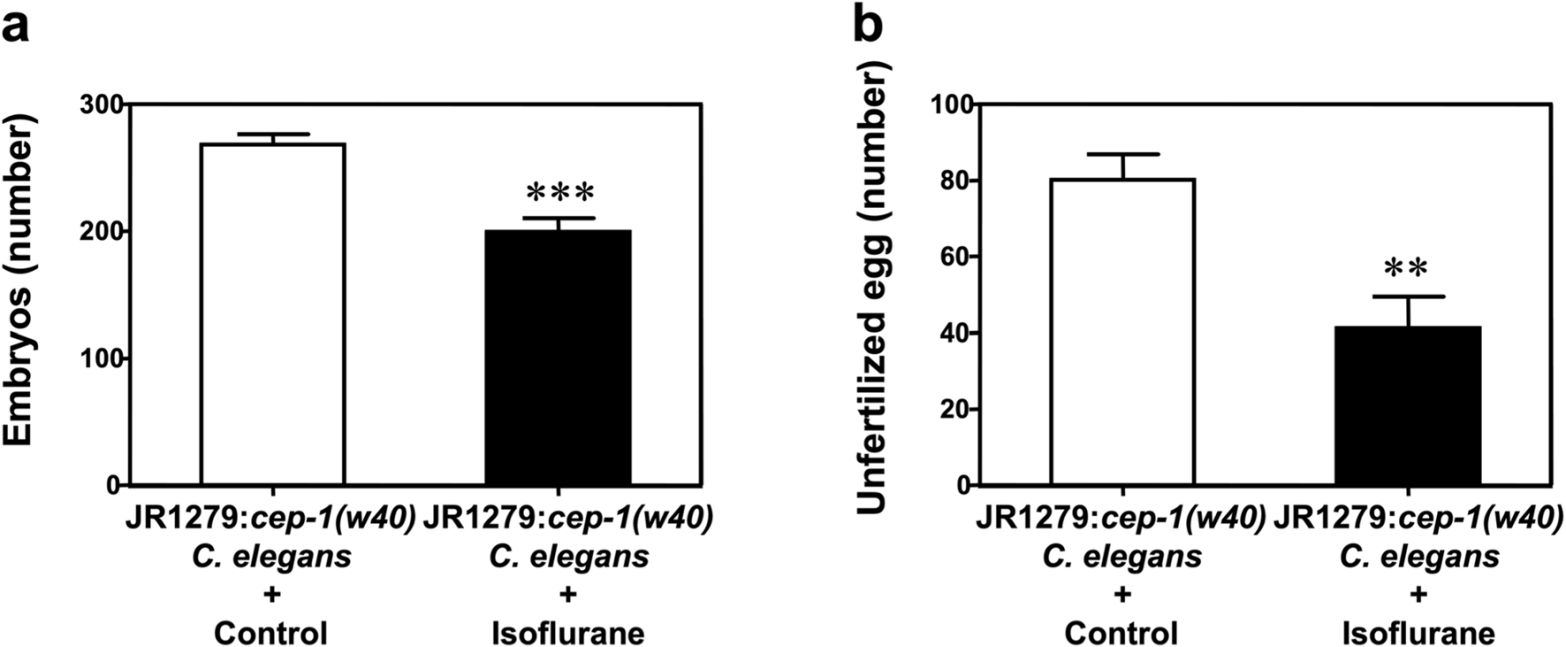
Isoflurane impaired oogenesis in *cep-1* knockout *C. elegans*. (**a**) Treatment with 7% isoflurane for 4 h decreased the number of embryos of *cep-1* knockout hermaphrodites. (**b**) Treatment with 7% isoflurane for 4 h decreased the number of unfertilized eggs of *cep-1* knockout hermaphrodites. All data are presented as mean ± S.D. N = 10 hermaphrodites in each group. JR1279: *C. elegans* strain *cep-1(w40)*. The Student's t-test was used to analyze the data presented in (**a**,**b**); the P values refer to the difference between the control condition and isoflurane. **P < 0.01, ***P < 0.001.

CEP-1 is a primordial *p53* family member^19^. When genotoxic stress induces the expression of DNA checkpoint proteins (such as MRT-2, HUS-1, and CLK-2), CEP-1 is activated to trigger DNA damage-associated germ cell apoptosis^18^. To investigate whether isoflurane impairs oogenesis through DNA damage-induced germ cell apoptosis, we used the *cep-1* knockout mutant strain JR1279 in fertilization assays. After exposure to the isoflurane treatment, the number of embryos (Fig. 2a) and unfertilized eggs (Fig. 2b) produced by JR1279 hermaphrodites significantly decreased compared to the control condition.

### Isoflurane increases ROS generation and impairs oogenesis via an *abl-1*-associated mechanism

**Figure 3:**
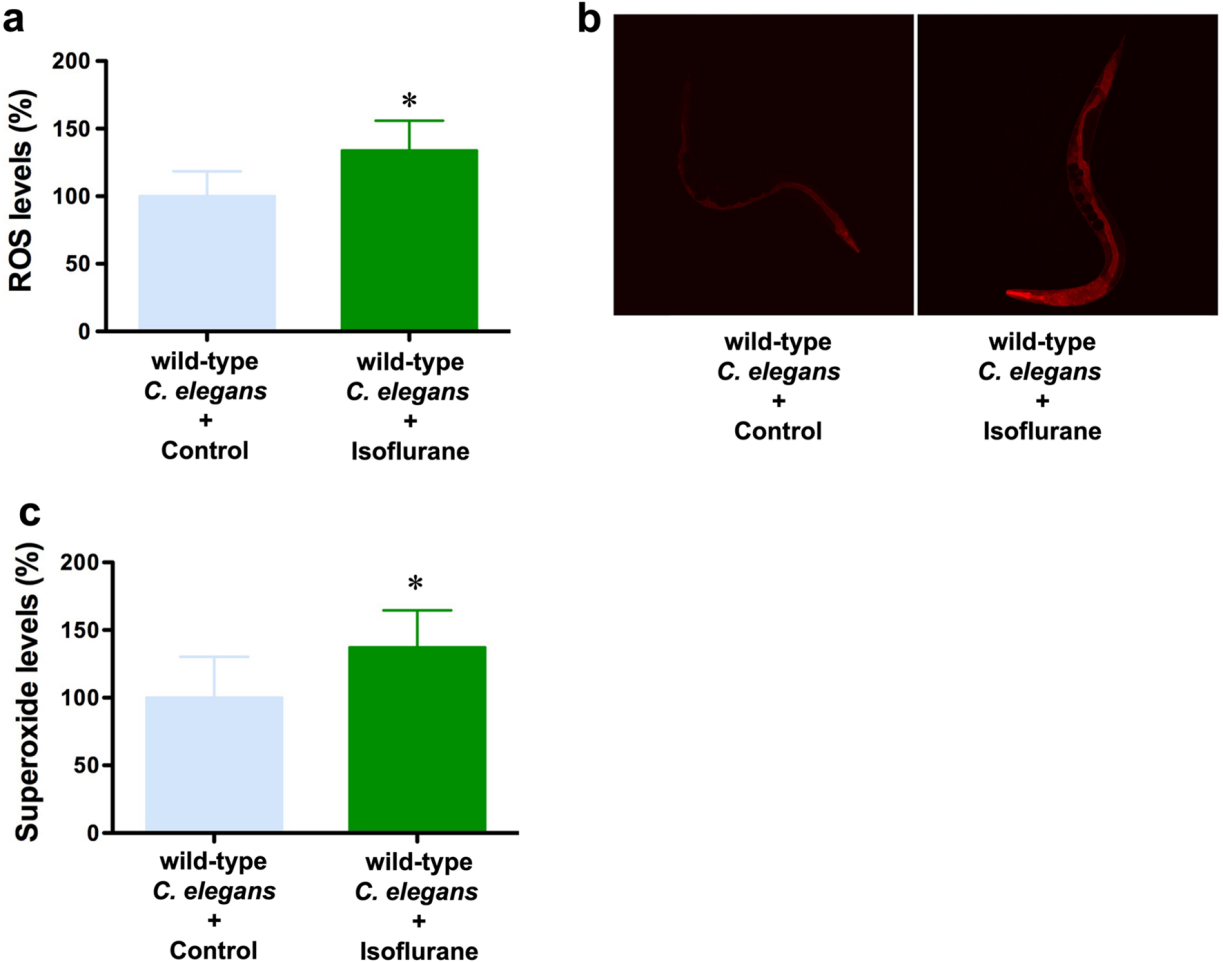
Isoflurane increased ROS levels in wild-type *C. elegans*. (**a**) Isoflurane increased the levels of ROS in *C. elegans* as compared to the control condition. (**b**) Superoxide staining by MitoSOX shows an elevated level of superoxide of *C. elegans* following the treatment with isoflurane and control conditions. (**c**) The quantification of the staining showed that the isoflurane treatment (green bar) increased the level of superoxide compared to the control condition (blue bar) in *C. elegans*. All data are presented as mean ± S.D. N = 10 hermaphrodites in each group. The Student's t-test was used to analyze the data presented in (**a**,**c**); the P values refer to the difference between the control condition and isoflurane. * *P* < 0.05.

Isoflurane may induce oxidative stress by promoting ROS production^9,10^, which is inhibited by N-acetyl-cysteine (NAC)^20^. Thus, we assessed the effects of isoflurane on reactive oxygen species (ROS) levels in *C. elegans.* We found that the treatment of isoflurane (7% isoflurane for 4 h) significantly increased the amounts of ROS (Fig. 3a) and superoxide (Fig. 3b,c) as compared to the control condition.

**Figure 4:**
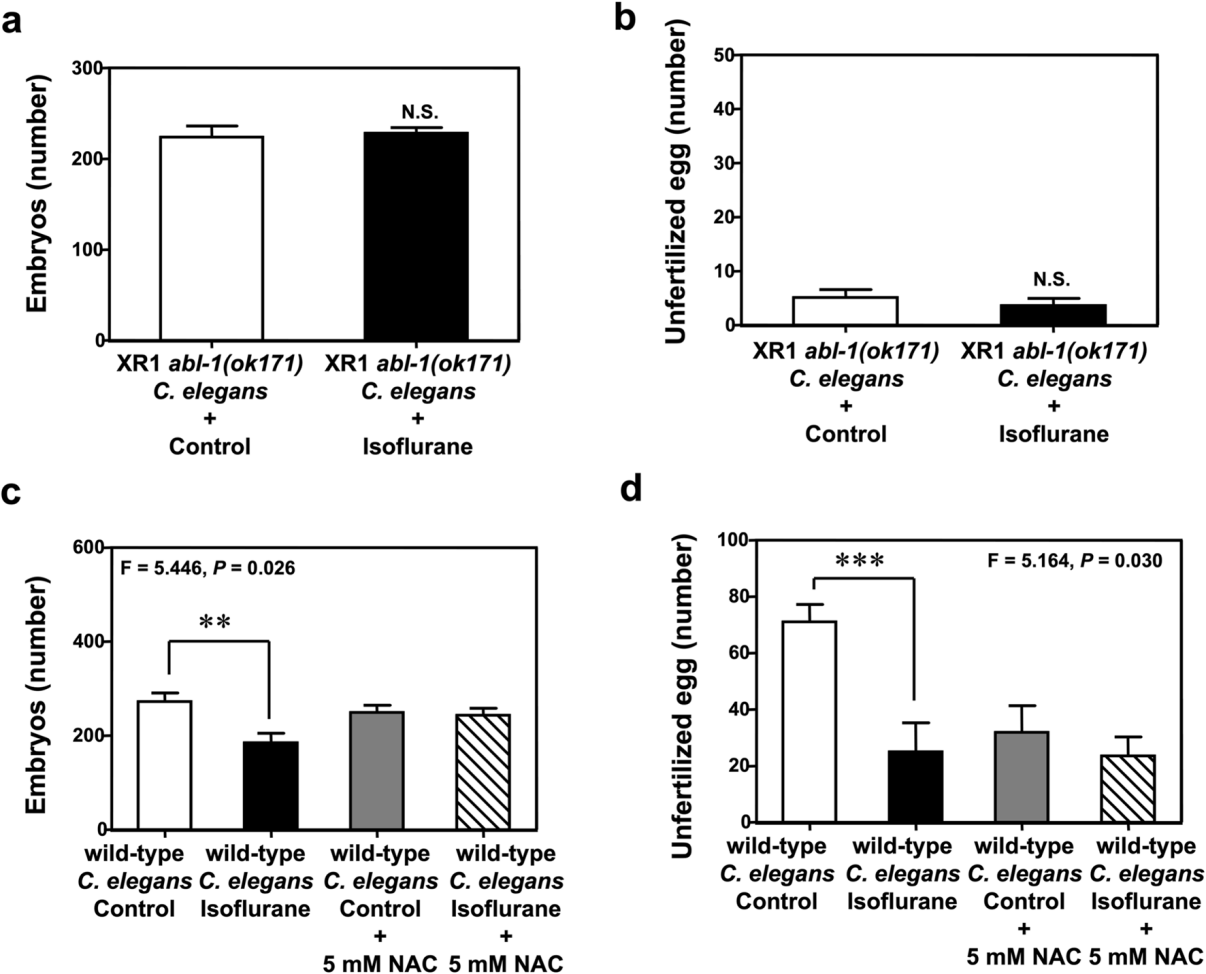
Isoflurane impaired oogenesis via an *abl-1-*associated oxidative stress-related pathway. (**a**) Treatment with 7% isoflurane for 4 h did not decrease the number of embryos produced by *abl-1* knockout hermaphrodites. (**b**) Treatment with 7% isoflurane for 4 h did not decrease the number of unfertilized eggs produced by *abl-1* knockout hermaphrodites. (**c**) Treatment with 5 mM N-acetyl-cysteine (NAC) in wild-type *C. elegans* mitigated the isoflurane-induced reduction in the number of embryos produced by N2 hermaphrodites. (**d**) Treatment with 5 mM NAC attenuated the isoflurane-induced reduction in the number of unfertilized eggs produced by N2 hermaphrodites. All data are presented as mean ± S.D. N = 10 hermaphrodites in each group. The Student's t-test was used to analyze the data presented in (**a**,**b**); the P values refer to the difference between the control condition and isoflurane. Two-way ANOVA and posthoc Bonferroni test was used to analyze the data presented in (**c**,**d**); the P values refer to the interaction of group (control condition versus isoflurane) and treatment (vehicle versus NAC), and the difference between each treatment. ** *P* < 0.01, *** *P* < 0.001. XR1: *C. elegans* strain *abl-1(ok171)*; *NAC* N-acetyl-L-cysteine.

To further explore the upstream mechanism by which isoflurane impaired oogenesis, we repeated the fertilization assay using an *abl-1* knockout mutant *C. elegans* strain, XR1. Isoflurane did not significantly decrease the number of embryos (Fig. 4a) or unfertilized eggs (Fig. 4b) in XR1 *C. elegans* compared to control. Finally, we used NAC as a ROS inhibitor and repeated the fertilization assay in wild-type *C. elegans*. We found that NAC attenuated the effects of isoflurane on the number of embryos (Fig. 4c) and unfertilized eggs (Fig. 4d).

### Mating with male *C. elegans* does not attenuate the isoflurane-induced changes in oogenesis

**Figure 5:**
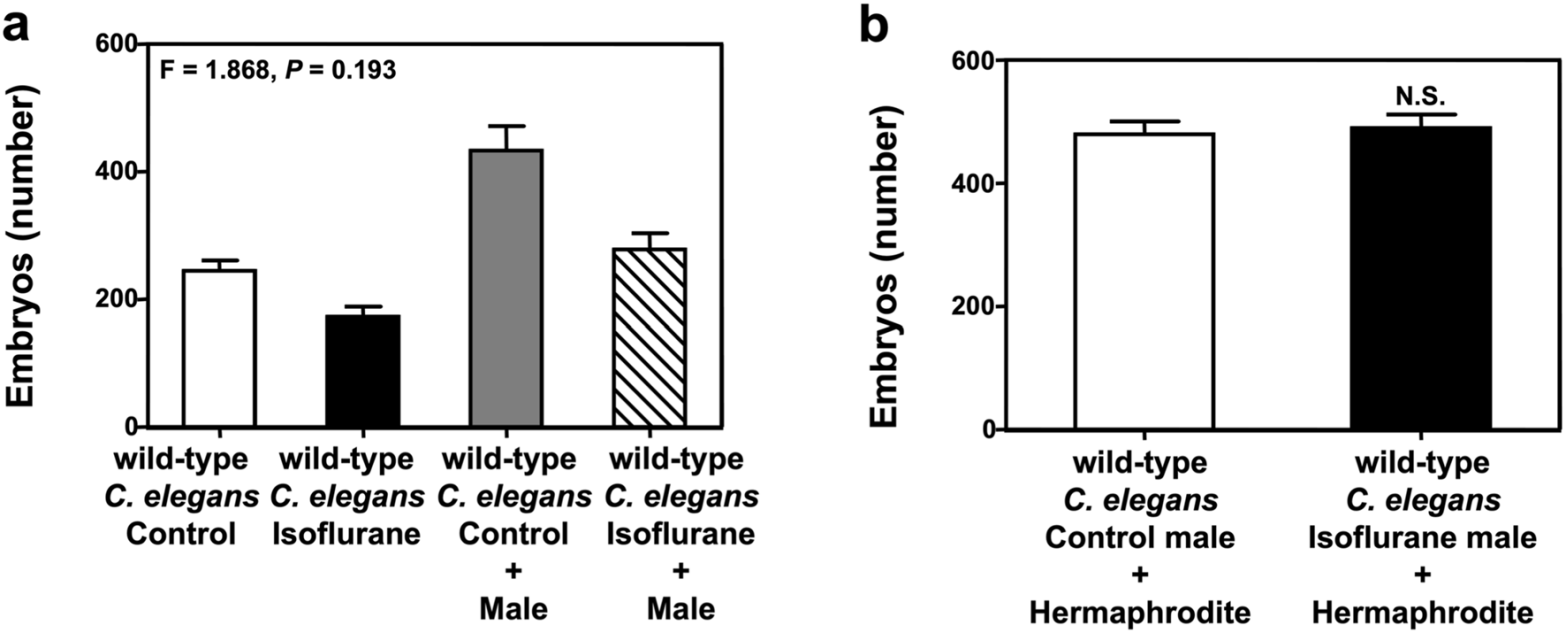
Mating with male *C. elegans* did not attenuate the isoflurane-induced changes in oogenesis. (**a**) Wild-type N2 hermaphrodites were treated with 7% isoflurane for 4 h. Then, five male *C. elegans* were transferred into each plate for mating with one hermaphrodite for 12 h. The number of embryos was significantly lower in the isoflurane group than the control group with or without mating with male *C. elegans*. (**b**) Five male *C. elegans* per plate were treated with 7% isoflurane, and then every five male *C. elegans* mated with one hermaphrodite for 12 h. There was no significant difference in the number of embryos between the control and isoflurane groups. All data are presented as mean ± S.D., N = 10 hermaphrodites in each group. Two-way ANOVA and posthoc Bonferroni test was used to analyze the data shown in (**a**); the P values refer to the interaction of group (control condition versus isoflurane) and treatment (with versus without male *C. elegans*). The Student's t-test was used to analyze the data presented in (**b**); the P values refer to the difference between the control condition and isoflurane.

The germ cells of hermaphrodites can produce about 150 sperm per gonad arm at the L4 stage^21^. Reduction or dysfunction of sperm can also contribute to embryo death. Therefore, we used male *C. elegans* to study the possibility that the isoflurane-induced impairment of oogenesis was due to spermatogenesis. Five male *C. elegans* were transferred into each plate for mating with one hermaphrodite for 12 h, followed by the fertilization assay. Mating with male *C. elegans* increased the number of embryos but did not significantly mitigate the isoflurane-induced reduction in the number of embryos (Fig. 5a). Moreover, synchronized L4 males were treated with isoflurane and then mated with hermaphrodites. The number of embryos remained unchanged between the control condition and isoflurane treatment (Fig. 5b).

## Discussion

We set out to assess whether the anesthetic isoflurane could impair oogenesis in *C. elegans* and reveal the underlying mechanism. We demonstrated that isoflurane could impair oogenesis potentially via stress-associated and *abl-1* associated germ cell apoptosis. Isoflurane did not appear to impair oogenesis in *C. elegans* via DNA damage-associated germ cell apoptosis. Finally, ROS inhibitor NAC attenuated the isoflurane-induced impairment of oogenesis. For the first time, these data reveal that isoflurane could affect fecundity by impairing oogenesis through, at least partially, oxidative stress- and germ cell apoptosis-associated mechanisms.

Importantly, these findings have established a system in *C. elegans*, generated hypotheses, and proved a general concept that anesthetic isoflurane could affect fecundity by impairing oogenesis in *C. elegans*. These efforts will likely lead to more research in anesthesia toxicity and, in particular, about anesthesia and fecundity in the future.

The life cycle of *C. elegans* is relatively short. It takes only 55 h for an N2 hermaphrodite to grow from an egg to an adult at 22 °C. When there is no male mating, one hermaphrodite will lay about 280 eggs^22^. This makes *C. elegans* an excellent experimental model to study oogenesis and the reproductive system. The egg-laying apparatus of *C. elegans* consists of the uterus, uterine muscles, vulva, vulva muscles, and local neuropil formed by egg-laying neurons. Since no behavioral abnormalities were observed and the worms treated with isoflurane could still ovulate embryos normally without exception (data not shown), it is unlikely that isoflurane treatment caused dysfunction of the egg-laying apparatus.

Notably, the gonad of hermaphrodite produces sperm cells during L4, and with the transition to adult, it switches to generate oocytes. In the present study, we treated the *C. elegans* with isoflurane during L4. Therefore, it is unlikely that the treatment with isoflurane in L4 can affect total oogenesis, which has not "officially" started yet in L4. However, it is conceivable that isoflurane treatment in L4 could impair the early-stage changes in oogenesis. Taken together, the observed reduction of unfertilized eggs after isoflurane treatment suggests that isoflurane may only impair certain parts of oogenesis but not the total oogenesis. Further studies to determine the effects of isoflurane on oogenesis in *C. elegans*, including oocyte maturation and embryonic viability, are warranted in the future.

The treatment of isoflurane in adult phase induced oocytes engulfed for resorption in *C. elegans* (Fig. 1c). But the treatment of isoflurane in L4 did not lead to oocytes engulfed for resorption in *C. elegans* (data not shown). These findings could be because L4 *C. elegans* are too young to develop apoptosis as oocytes engulf resorption. These data suggest that the findings from the study of isoflurane treatment during adult *C. elegans* can be used as a proxy that the treatment of isoflurane during L4 could induce apoptosis in *C. elegans*, pending further investigation.

These findings suggest that the treatment of anesthetics in a different stage of *C. elegans* (e.g., L4 versus adult) may generate different findings in apoptosis and oogenesis. This hypothesis is supported by the results from the studies of anesthesia-induced neurotoxicity. Specifically, Deng et al. demonstrated that susceptibility to the isoflurane-induced apoptosis could parallel to the peaks of neurogenesis, determined in different ages of young mice^23^.

Germ cells, but not somatic cells, can undergo DNA damage-associated or stress-associated apoptosis. Apoptotic germ cells after genotoxic insults are morphologically indistinguishable from physiologically apoptotic germ cells^12^. Thus, to further study the upstream mechanism of germ cell apoptosis, we used *cep-1* and *abl-1* mutant *C. elegans*. CEP-1 plays an essential role in DNA damage-induced germ cell apoptosis^19^. ABL-1 is involved in stress-associated germ cell apoptosis^15^. Our results showed that isoflurane could induce impairment of oogenesis in *C. elegans* via an *abl-1*-associated pathway. These data suggest that isoflurane impairs oogenesis via stress-associated but not DNA damage-associated germ cell apoptosis.

The cascade of apoptosis in *C. elegans* includes cell death abnormal 4 (CED-4), CED-9, and CED-3. During the apoptosis, egg-laying defective 1 (EGL-1) can bind to CED-9, which leads to a conformational change to release CED-4. The released CED-4 travels to the nucleus to activate CED-3, initiating apoptosis^24^. The findings that isoflurane did not decrease the number of embryos and unfertilized eggs in CED-3 knockout *C. elegans* suggest that it is likely that CED-3 contributes to the isoflurane-induced impairment of oogenesis in *C. elegans*. However, given that it was unknown whether the effects of isoflurane and CED-3 were additive, the mediated role of CED-3 on the isoflurane-induced impairment of parts of oogenesis was likely but not certain.

Oxidative stress is associated with low blastocyst rate, low fertilization rate, low cleavage rate, and high embryonic fragmentation^25^. In the present study, we found that the isoflurane treatment induced oxidase stress by increasing ROS and superoxide in *C. elegans*. The oxidative stress inhibitor NAC attenuated the effect of isoflurane on the number of embryos and unfertilized eggs. These data suggest a role for oxidative stress in isoflurane-induced impairment of oogenesis. NAC can mitigate the isoflurane-induced impairment of oogenesis in *C. elegans*.

*C. elegans* has two sexual forms: self-fertilizing hermaphrodites and males. Hermaphrodites are female, and their gonads temporarily produce sperm before they produce oocytes. Sperm stored in the gonads is limited, which is why one hermaphrodite can only produce ~ 300 offspring. When the hermaphrodite mates with males, it can have more than 1000 offspring^26^. In the final stage of our study, mating with male *C. elegans* did not significantly attenuate the isoflurane-induced reduction in the number of embryos. These findings suggest that the treatment of isoflurane in L4 in the present study does not significantly affect the sperm of *C. elegans*. However, we only used one concentration of isoflurane (7%) in the present study. Moreover, isoflurane was treated in *C. elegans* during L4, the proliferation stage of germ cell development in the distal gonad. Therefore, we cannot rule out that the sperm may still be affected under different conditions (e.g., treatment with a higher concentration of isoflurane or earlier exposure to isoflurane). Collectively, these results will promote more research, including qualitative and quantitative analysis of how active sperm are present in the isoflurane-treated *C. elegans,* to determine whether isoflurane can impair oogenesis by affecting sperm in *C. elegans*.

There are limitations in the present study. First, the germ cells were not isolated from somatic cells. Thus, we were unable to perform western blotting of CED-3, CEP-1, or ABL-1 proteins. Future studies should also include tissue-targeted RNAi to knock down gene expression to reveal further the role of CED-3, CEP-1, or ABL-1 in the isoflurane-induced oogenesis impairment in *C. elegans*. Second, we did not use Nomarski imaging or loss-of-function mutants for germ cell engulfment and clearance studies. We only used one knockout *C. elegans* per apoptosis signaling pathway in the present study. However, the main objective of the present study was to determine whether isoflurane could impair oogenesis in *C. elegans* via the apoptosis signaling pathway but was not to thoroughly assess the effects of isoflurane on apoptosis in *C. elegans*. Finally, we did not determine the direct effects of isoflurane on sperm, including analysis of active sperm in the isoflurane-treated *C. elegans*. Spermatogenesis creates functional sperm from an initially undifferentiated germ cell. Many factors, such as mutations that affect sperm meiosis, and cytoskeletal mutations, can impair spermatogenesis. Further experiments should be conducted to study the direct effect of isoflurane on sperm.

In conclusion, we established a system to investigate the effects of anesthesia on fecundity by looking at oogenesis in wild-type and mutant *C. elegans*. We found that isoflurane could impair oogenesis potentially through stress-associated and *abl-1*-associated germ cell apoptosis. Isoflurane-induced impairment of oogenesis was mitigated by oxidative stress inhibitor NAC, indicating that oxidative stress contributes to this process. Finally, mating with male *C. elegans* did not attenuate the isoflurane-induced changes in oogenesis, but it remains undetermined whether isoflurane could impair sperm in *C. elegans*. These findings will help generate further hypotheses to promote more research in anesthesia toxicity and fecundity.

## Materials and methods

### *C. elegans* strains

Wild type N2, JR1279: *cep-1(w40)*, MT1522: *ced-3(n717)*, XR1: *abl-1(ok171)*, and MD701: *bcls39 V C. elegans* strains were obtained from the Caenorhabditis Genetics Center (Minneapolis, MN). All *C. elegans* were grown on agar plates spread with *Escherichia coli* OP50 and maintained at 22 °C.

### Anesthesia

For fertilization studies and fluorescence microscopy assays, 20 adult hermaphrodites were picked per plate and incubated for 2 h to allow them to lay eggs. Usually, *C. elegans* progress through four larval stages (L1, L2, L3, and L4) and reach the L4 stage at 37 h and the adult stage at 55 h after eggs are laid^22^. *C. elegans* were randomly assigned to isoflurane and control groups. L4 larvae assigned to the isoflurane group were exposed to isoflurane (7%) plus air for 4 h at 22 °C in a 20 × 20 × 7-cm induction chamber. Notably, 7% isoflurane is the EC50 for causing immobility in *C. elegans*^27^. The isoflurane flow rate was 2 L/minute for the first 3 min to induce anesthesia and then 1 L/minute to maintain anesthesia. The control group was exposed to air only for 4 h at an identical flow rate and temperature. Concentrations of isoflurane were measured continuously by a gas analyzer (Ohmeda, GE Healthcare, Tewksbury, MA).

### Fertilization assay and reactive oxygen species (ROS) inhibitor treatment

After treatment with isoflurane or control condition for 4 h, each L4 larva was transferred to a new plate (3.5 cm in diameter). These L4 larvae would grow into adults after approximately 13 h and then begun to lay eggs. We transferred each of them to a new plate once daily, and then we counted the progeny (both the eggs and hatched larvae) of the treated *C. elegans* on the previous plate as the number of embryos until there were no more eggs laid on the plate. Given that we did not count the cells in the gonad, we used the name of unfertilized eggs to describe the effects of isoflurane on oogenesis. When the worms reached adulthood day 3, they might run out of sperm, and thus the oocytes could not be fertilized. The number of unfertilized eggs was then counted. Embryos, unfertilized eggs, and larvae were quantified by an observer blinded to treatments, and the counting was repeated at least three times. Some *C. elegans* were treated with ROS inhibitor N-acetyl-cysteine (NAC, 5 mM) as previously described^28^. Specifically, 50 g NAC (Sigma-Aldrich, Milwaukee, WI) was added to the nematode growth medium (NGM) before preparing for a final concentration of 5 mM. N2 hermaphrodites were transferred to NGM or NGM plus 5 mM NAC plates to lay eggs. When the eggs hatched and grew into L4 larvae, they were treated with isoflurane or control air for 4 h, followed by the fertilization assay.

### Measurement of ROS

Intracellular ROS in *C. elegans* was measured using DCFH-DA (Cell Biolabs, San Diego, CA). At the end of isoflurane anesthesia or control condition, the *C. elegans* were collected into PBS with 1% Tween20 in Eppendorf tubes and homogenized by Branson Sonifier 450 Digital Ultrasonic Homogenizer. The homogenization was transferred into 96-well plated and incubated with 20 μM DCF-DA in PBS at 37 °C for one h away from light. Finally, the fluorescence was read with a SpectraMax M5 microplate reader (Molecular Devices Co., Sunnyvale, CA) at 480 nm/530 nm.

### Measurement of superoxide

Superoxide in *C. elegans* was measured by using MitoSOX Red (Invitrogen, Carlsbad, CA). Adult *C. elegans* were transferred to M9 buffer containing 5 µM MitoSOX, then received isoflurane anesthesia for 4 h. After anesthesia, the *C. elegans* were transferred to new NGM plates to clear their intestinal tract of residual dye. Then *C. elegans* were paralyzed on the slides in 8 µl of 5% lidocaine. The fluorescent images were taken using a Nikon Eclipse Ti confocal microscope (Nikon, Tokyo, Japan) with the same exposure settings. The intensity of MitoSOX fluorescence accumulated in the whole body of individual *C. elegans* was quantified by using ImageJ.

### Fluorescence microscopy to detect cell corpses

The MD701 strain of *C. elegans* expresses P_lim-7_ced-1:GFP combined with protein CED-1. During germ cell apoptosis, the GFP creates a halo around dying cells^29^. MD701 *C. elegans* were synchronized by egg-laying. When *C. elegans* reached adulthood day 1 (as L4 larvae were too young to observe germ cell apoptosis), they were treated with isoflurane or control air using the same method described above. Then adult *C. elegans* at day 1 was mounted on agar pads containing a drop of 40 mM NaN_3_ dissolved in sterilized M9 buffer (3 g KH_2_PO_4_, 6 g Na_2_HPO_4_, 5 g NaCl, 1 mL of 1 M MgSO_4_). Dead cells expressing GFP in each gonad arm were observed under a fluorescence microscope at 40X magnification, and the number of dead cells was counted in a blind manner. Images were obtained using a Nikon Eclipse E600.

### Fertilization assay after crossing with males

Heat shock and backcrossing were used to obtain male *C. elegans* (Sulston J and Hodgkin J, methods in the nematode *C. elegans*). When we obtained 100 N2 (wild type) male *C. elegans*, we transferred them to a plate and crossed them with the isoflurane- or control-treated N2 L4 hermaphrodites at a 5:1 ratio of males to hermaphrodites. We set the mating duration to 12 h because L4 hermaphrodites grow into the adulthood stage and start to lay embryos after 18 h. Treated hermaphrodites obtained abundant sperm from the males by mating. Hermaphrodites were then transferred to a new plate (one hermaphrodite per plate), and the number of embryos laid by each hermaphrodite was quantified until there were no more embryos laid on the plates. We also picked 10 adult males and 10 adult hermaphrodites per plate and mated them for 2 h to obtain synchronized N2 L4 males. Synchronized N2 L4 males were then treated with isoflurane or control conditions as described above. Finally, we crossed isoflurane- or control-treated synchronized males with synchronized L4 stage hermaphrodites for 12 h at a 5:1 ratio of males to hermaphrodites. Hermaphrodites were transferred to a new plate to quantify the number of embryos laid.

### Statistical analysis

All data are expressed as mean ± standard deviation (S.D.). For the fertilization assay, we performed a power analysis based on data obtained in preliminary experiments. Assuming a mean difference of 60 (280 versus 220) embryos laid by N2 hermaphrodites, SD of 75 in the control arm, and SD of 62 in the anesthesia arm, a sample size of 10 per group, provides 90% power to detect a difference in fertilization changes using a two-sided t-test with 5% type I error. Interaction between NAC and group factors in two-way ANOVA was used to analyze the difference in numbers of embryos and unfertilized eggs between *C. elegans* in control and isoflurane-treated groups. Student's t-test with Bonferroni correction was used to compare the numbers of embryos and unfertilized eggs between control and anesthesia groups with NAC treatment. The Student's t-test was used to determine the difference in dead cells between isoflurane and control groups during the fluorescent assay to confirm that isoflurane induces germ cell apoptosis. Finally, the interaction between male rescue effects and group factors was analyzed using two-way ANOVA. The Student's t-test was used to determine the difference in ROS amounts between isoflurane and control conditions. There were no missing fertilization assay variable data. Hypothesis testing was two-tailed. *P* values of < 0.05 were considered statistically significant. SAS software (Cary, NC) and Prism 9 software (La Jolla, CA) analyzed the data.
